# Discerning cognitive domains through online assessment in aging participants in Norwegian Cohort 50+

**DOI:** 10.1038/s41598-025-26437-8

**Published:** 2025-11-27

**Authors:** Anastasia Ushakova, Anne Corbett, Clive Ballard, Jon Arild Aakre, Martha Therese Gjestsen, Dag Aarsland, Ingelin Testad

**Affiliations:** 1 Centre for Age-Related Medicine-SESAM, Stavanger University Hospital, Stavanger, Norway; 2 Department of Health and Community Sciences, Faculty of Health and Life Sciences, University of Exeter, Exeter, UK; 3 Department of Clinical Biosciences, Faculty of Health and Life Sciences, University of Exeter, Exeter, UK; 4 Department of Clinical Medicine, University of Bergen, Bergen, Norway; 5 Department of Psychological Medicine, Institute of Psychiatry, Psychology and Neuroscience, King’s College London, London, UK

**Keywords:** Geriatrics, Public health, Quality of life, Lifestyle modification

## Abstract

Cognitive decline is a significant aspect of aging, varying across domains such as executive function, working memory, and attention. Identifying modifiable risk factors and their domain-specific effects is crucial to mitigating age-related decline. Advances in online cognitive assessments facilitate large-scale studies across diverse populations, offering new opportunities for understanding cognitive aging. This analytical study utilized baseline data from 3215 participants in the PROTECT Norge cohort (aged 50+). Participants completed six cognitive tasks, alongside questionnaires on lifestyle, health, and dementia risk factors. Multivariate analyses, including exploratory factor analysis (EFA) and Independent Component Analysis (ICA), identified latent cognitive constructs. Regression models assessed the effects of aging and risk factors on cognitive performance, adjusting for demographic variables. Two latent cognitive constructs—executive function and working memory—were identified, alongside error-derived components reflecting focused attention and short-term spatial memory. Older age was associated with lower scores in all domains, with the largest effect observed for executive function [− 0.06 standard deviations (SD) per year], followed by working memory (− 0.05 SD per year), focused attention (− 0.03 SD per year), and short-term spatial memory (− 0.01 SD). Known dementia risk factors, such as stroke, smoking, and high blood pressure, significantly impaired cognitive domains, while higher education and employment provided protective effects. Moderate alcohol consumption correlated positively with cognitive outcomes, though excessive consumption had an adverse effect. The study highlights domain-specific associations between age and cognitive functions with age and the varied impact of dementia risk factors. These results highlight the value of multidimensional cognitive assessments in improving our understanding of age-related cognitive changes and the factors that influence them.

**Supplementary Information:**

The online version contains supplementary material available at 10.1038/s41598-025-26437-8.

## Introduction

Western populations are experiencing a demographic shift toward older age^1^, resulting in an increased prevalence of age-related conditions, particularly dementia.

Cognitive decline, a recognized aspect of aging, is often seen as a precursor to more severe conditions such as dementia. Various risk factors, including genetics, lifestyle, and health conditions, contribute to this process, with some individuals experiencing faster rates than others. Identifying these factors is crucial, as interventions aimed at modifiable risk factors could slow cognitive decline and potentially delay or prevent dementia onset.

The ageing process affects cognitive functions at varying rates; for instance, processing speed and working memory often decline earlier and more rapidly than crystallized abilities such as vocabulary and general knowledge^2^. This heterogeneity is likely related to differential neurobiological changes across cognitive domains, as the aging brain undergoes changes at every level, from molecules to morphology^3–6^. Identifying specific health and lifestyle factors associated with accelerated decline in certain cognitive domains is crucial for developing targeted interventions to support cognitive health in aging populations.

Recent technological advances, have enabled large-scale administration of cognitive assessments via online platforms, facilitating research on cognitive aging across different populations. This study uses the data from the PROTECT Norge study, an online cognitive test battery administered to participants aged 50 and older (https://www.protect-norge.no/). The tests included in this battery were designed to assess different abilities of cognitive functioning. Cognitive capacity itself can be conceptualized as comprising various domains, each representing distinct aspects of cognitive functioning^7^. Accordingly, scores obtained from multiple disparate cognitive tasks can be used to evaluate the strength of domains of cognition.

Several studies have attempted to analyze cognitive domains using computerized cognitive batteries^8^, such as computerized ARMADA study^9^ or Cogstate Brief Battery^10^, aiming to enhance understanding of healthy aging. Our study builds on this research by analyzing new data from a Norwegian cohort and applying advanced multivariate analysis methods. Given the number of individual tasks in the PROTECT Norge computerized cognitive test battery, we applied a data reduction technique to identify underlying latent factors representing broader cognitive domains. This approach enhances the reliability of measurement within each domain, reduces random measurement error, and facilitates more parsimonious analysis and interpretation of the cognitive data. By summarizing task-level performance into cognitive domains, we are better able to examine associations with participant characteristics and risk factors at a domain level rather than relying solely on individual task scores.

Our study has three primary objectives: to identify the latent cognitive domains measured by the PROTECT Norge battery, to compare effects of age across these cognitive domains, and to evaluate the impact of dementia risk factors^11,12^ on cognitive performance. These aims collectively seek to enhance understanding of cognitive aging and inform future strategies for preserving cognitive health across varying cognitive functions.

## Materials and methods

### Study design

This is an analytical study using baseline data from an ongoing digital cohort study.

### Data collection

The participants were recruited via the online platform PROTECT Norge (https://www.protect-norge.no/). The study was approved by the Regional Committee for Medical and Health Research Ethics, West (Ref #2019/478), and conducted in accordance with the principles of the Declaration of Helsinki. Written informed consent was obtained from the participants for their participation in this study.

The data were collected during the period between September 2020 and September 2022. The data from N = 3215 participants contained the scores of six cognitive tests which are presented in the next paragraph. In addition, the participants completed questionnaires describing their lifestyle, medical history, and mental health, as shown in Table 1. The characteristics were selected based on the major modifiable dementia risk factors outlined by the Lancet Commission^11^, using those that were available in PROTECT Norge dataset. Depression was assessed using the validated nine-item Patient Health Questionnaire (PHQ-9)^13^; participants with ‘moderately severe’ or ‘severe’ scores were classified as depressed. Higher education was recorded if the participant had attended high school or university—regardless of whether the program was completed—or held a doctoral degree.

### Cognitive tests

Cognitive assessment was performed using a battery of six disparate tests aimed to measure different cognitive functions^14^.

Digit Span Memory Test: evaluates verbal working memory;

Paired Associate Learning: measures visual episodic memory and learning;

Self-Ordered Search Task: assesses spatial working memory;

Grammatical Reasoning Task: evaluates verbal reasoning;

Switching Stroop Test: measures selective attention and processing speed;

Trail Making A and B Test: assesses visual attention, mental flexibility, processing speed, and motor speed.

Four of the cognitive tests used—Paired Associate Learning, Self-Ordered Search, Digit Span, and Verbal Reasoning—are included in the validated FLAME battery^15^, which has demonstrated sensitivity to cognitive impairment, responsiveness to change, and good validity^15,16^. Although the Stroop and Trail Making tasks are not part of FLAME, they are widely used, well-established neuropsychological measures with robust normative data^17^. A short presentation of the tests and details of the computation of the scores are provided in the Supplementary Table 1.

We used the total scores of each test for the main analysis. To extract more information from the available data, we also analyzed the error scores. For the Digit Span, Paired Associates Learning and Self-Ordered‐Search tests, progression to a new level of complexity is only allowed if no more than three consecutive errors are made; otherwise, the test is terminated. As a result, a high error score can occur even if the total score is high. Therefore, for these three tests, we used error rates, defined as the number of errors divided by the total score. For all other tests, the raw error scores were used.

### Statistical analysis

The total scores and numbers of errors were used for multivariate analyses. The total scores had all distributions close to normal, with the exception of Trail Making test. The total scores of Trail Making test had a skewed distribution and were transformed by − log(x) transformation for further analysis to ensure that the distribution was close to normal. All the error scores had skewed distributions.

Each test included one to three repeats. The dimension reduction process used the average scores over the available repetitions. Eigen value analysis^19^ suggested a two-factor model.

Exploratory factor analysis (EFA) with *oblimin* rotation was used to estimate two latent factors summarizing the multivariate data of cognitive total scores. A source separation method, Independent Component Analysis (ICA) by entropy maximization^20^, was used to extract components representing two presumably distinct functional brain networks underlying cognitive domains associated with task performance.

The estimated latent construct scores obtained via the EFA and ICA methods were then analyzed by linear regression models to evaluate associations with age and assess the impact of the known dementia risk factors. Each latent construct score transformed to *Z*-scores served as the outcome variable, and the risk factors (one at a time) were included as the main effect; all models were adjusted for age, gender, higher education status and the number of times the test battery was repeated.

In addition, to quantify the effect of each year of age, scores for each latent construct were standardized to a scale of 0–100, where 0 represents the population minimum value and 100 represents the maximum value. Each model was adjusted for gender, whether participant had a higher education, and the number of times the test battery was completed.

No correction for multiple testing was performed due to the exploratory nature of this study. All *P*-values were two-sided, with a significance level of 0.05. In addition, given the four outcome variables representing the cognitive domains, we applied a more stringent significance level of 0.01, which provides a practical approximation to a Bonferroni adjustment for multiple comparisons across these outcomes. All the statistical analyses were performed using R Project for Statistical Computing, version 4.3.3. EFA was performed using package *psych*, version 2.4.3. ICA was performed using package *ica*, version 1.0-3.

## Results

### Completion of questionnaires

During the first two years of our study, 3215 participants completed the baseline cognition evaluation. All the participants provided basic demographic data, 85.8% completed the lifestyle questionnaire, 86.0% completed the medical history questionnaire and 77.0% provided information on their mental health.

### Baseline characteristics

The participants in this study included all individuals who registered for the PROTECT Norge study by November 2022 and who completed the cognitive assessment. Approximately 75% of the participants were women, and 75% were younger than 70 years old. Other characteristics are detailed in Table 1. Known risk factors for dementia^11^, such as diabetes, stroke^16^, social isolation, and physical inactivity, were underrepresented, with prevalences of 3.9%, 2.4%, 2.7%, and 8.9%, respectively. Additionally, less than 6% of the participants were current smokers.

**Table 1:** Characteristics of the study cohort.

Characteristic, *n* = 3215	Statistic	Missing values, *n* (%)
Age, median (IQR)	64.0 (57.9, 69.6)	0 (0.0)
Female, n (%)	2394 (74.5)	0 (0.0)
Higher education, n (%)	2504 (77.9)	0 (0.0)
Marital status, n (%)		23 (0.7)
Married/ civil partnership/ co-habiting	2290 (71.7)	
Widowed/ separated/ divorced/ single	902 (28.3)	
Working (full-time or part-time), n (%)	1609 (50.2)	8 (0.2)
Has a doctor ever given you a diagnosis of, or told you that you have…		
Diabetes, n (%)	107 (3.9)	458 (14.2)
High blood pressure, n (%)	656 (23.8)	458 (14.2)
Heart disease, n (%)	187 (6.8)	458 (14.2)
Stroke, n (%)	67 (2.4)	458 (14.2)
Have you ever experienced a head injury where you lost consciousness?, n (%)	407 (14.7)	450 (14.0)
Do you have problems with your hearing?, n (%)	818 (29.6)	450 (14.0)
Depression, n (%)	510 (20.6)	739 (23.0)
Isolation (no confiding relations since 16 years old), n (%)	64 (2.7)	882 (27.4)
Have you done any physical activity lasting at least 20 min that has left you out of breath in the last month?, n (%)	2512 (91.1)	458 (14.2)
How often do you normally have a drink of something with alcohol in?, n (%)		458 (14.2)
Never	165 (6.0)	
Less than once a month	432 (15.7)	
Less than once a week	656 (23.8)	
At least weekly	1504 (54.6)	
Drinking more than 2 drinks containing alcohol on a typical day when you are drinking, n (%)	712 (31.1)	924 (28.7)
Smoking		458 (14.2)
Never smoked	1138 (41.3)	
Current smoker	153 (5.5)	
Stopped smoking	1466 (53.2)	
BMI		456 (14.2)
Healthy (18.5–24.9)	1277 (46.3)	
Underweight (< 18.5)	24 (0.9)	
Overweight (25.0–29.9)	1058 (38.3)	
Obese (≥ 30)	400 (14.5)	
Self-reported sleep dissatisfaction, n (%)	713 (26.5)	529 (16.5)
Total scores	Mean ± SD	
Digit Span Memory Test	6.3 ± 2.5	
Paired Associate Learning	3.6 ± 1.0	
Self-Ordered‐Search task	6.5 ± 2.5	
Grammatical Reasoning task	24.5 ± 10.1	
Stroop test	34.0 ± 14.4	
Trail Making test (min)	1.2 ± 0.5	
Error scores	Mean ± SD	
Digit Span Memory Test	4.3 ± 1.2	
Paired Associate Learning	4.1 ± 1.0	
Self-Ordered‐Search task	1.0 ± 1.8	
Grammatical Reasoning task	4.5 ± 3.6	
Stroop test	11.0 ± 6.3	
Trail Making test	2.7 ± 4.9	
Maximum number of repeats	1.8 ± 0.8	

### Dimension reduction and source separation

The total scores data underwent dimension reduction using exploratory factor analysis (EFA) to achieve a two-dimensional solution. EFA has shown a reasonable fit, with a root mean square error of approximation (RMSEA) of 0.03 (90% CI 0.016–0.047) and a Tucker Lewis index of 0.98. Furthermore, the error scores data were subjected to blind source separation via Independent Component Analysis (ICA), aiming for a two-component solution. The loadings for each method are presented in Table 2.

**Table 2:** The loadings of each original variable on the identified latent constructs are presented. Exploratory factor analysis (EFA) was performed for the total scores of the cognitive tests, while independent component analysis (ICA) was used for the error scores. Loadings with absolute values greater than 0.3 are highlighted in bold.

	Cognitive test	Loadings for total scores		Loadings for error scores	
		EFA		ICA	
		F_1_ executive function	F_2_ working memory	ICA_1_ focused attention	ICA_2_ short-term spatial memory
Working memory	Digit span memory test	0.09	**0.41**	**− 0.32**	0.07
	Paired associate learning	0.18	**0.38**	**− 0.30**	0.28
	Self-ordered‐search task	0.23	0.28	− 0.01	− 0.92
Executive function	Grammatical reasoning task	**0.60**	0.14	**− 0.39**	0.00
	Stroop test	**0.67**	− 0.17	**− 0.36**	− 0.21
	Trail making test	**0.60**	0.17	**− 0.33**	− 0.09
	Explained variance	0.23	0.10	0.28	0.17

On the basis of the specified objectives outlined in “Cognitive tests”, it is understood that the Digit Span Memory test, Paired Associate Learning, and Self-Ordered-Search task were designed to assess working memory, while the Grammatical Reasoning task, Stroop test, and Trail Making test were intended to evaluate Executive Function/Cognitive Control domain, reflecting abilities related to task-switching, inhibition, and reasoning, even though optimal performance on any given test typically necessitates effective coordination among all cognitive systems. The factor loadings for the EFA solution, F_1_ and F_2_ in Table 2, suggest the interpretation of these latent constructs as mainly executive function for F_1_ and working memory for F_2_.

The error scores data exhibited skewed distributions, and ICA was employed. ICA excels in separating multivariate signals (error scores) into additive, independent components, focusing on underlying hidden sources.

Based on the loading scores in Table 2, the first independent component (ICA_1_) can be interpreted as representing focused attention intensity, as it exhibits meaningful loadings across all tasks’ error scores, except for the Self-Ordered Search task. This exception aligns with the nature of the Self-Ordered Search task, which demands sustained rather than focused attention. The second component (ICA_2_) primarily loads on the error rate scores from the Self-Ordered Search task, suggesting that it reflects spatial short-term memory capacity. The opposite loading direction of ICA_2_ on the Paired Associates Learning task may indicate that the latter relies more on spatial long-term memory, with potential interference between these two memory systems as they may share some underlying neural mechanisms.

### Correlation analysis and age effects of latent constructs

The Pearson correlation coefficient between the latent construct for executive function (F_1_) and focused attention (ICA_1_) was 0.63, and that between working memory (F_2_) and focused attention (ICA_1_) was 0.61. In contrast, the correlation between executive function (F_1_) and short-term spatial memory (ICA_2_) was 0.04, and that between working memory (F_2_) and short-term spatial memory (ICA_2_) was 0.13. Additionally, the correlation between executive function (F_1_) and working memory (F_2_) was notably high at 0.83.

Among the cognitive domains assessed, executive function showed the largest effect of age (Table 3), with β = − 0.92 (95% CI − 0.98 to − 0.87) percentage points per year. This was followed by working memory, with β = -0.75 (95% CI − 0.81 to − 0.69) percentage points per year of age. Focused attention showed a more moderate effect of age, with β = − 0.36 (95% CI − 0.41 to − 0.31) percentage points per year. The effect of age on short-term spatial memory was the smallest, with β = − 0.05 (95% CI − 0.09 to − 0.01) percentage points per year.

### Effects of dementia risk factors on cognitive performance

In this section, we investigated the influence of established risk factors associated with dementia on cognitive function. The outcome variables were the latent constructs derived from EFA applied to the total scores and ICA applied to the error scores. Standardized estimates from the regression analyses are presented in Table 3. Standardized outcome variables were used to facilitate comparisons across predictors. For such outcomes, the regression coefficients indicate how many standard deviations the outcome will change for each one-year increase in age or for each category shift in a categorical predictor, simplifying interpretation and comparison.

For the age variable, the annual changes in percentage points were also presented.

**Table 3:** Predictors of cognitive latent constructs. The latent constructs scores were averaged across the number of repetitions for each participant. The regression coefficients estimate the change in the outcome, measured in standard deviations (SD), per year for age or between categories for categorical predictors. The regression coefficients are presented as β (standard error), with *P*-values denoted by asterisks: *p < 0.05, **p < 0.01, ***p < 0.001. All estimates are adjusted for age, gender, whether participant has higher education, and the number of times evaluation was repeated.

Latent construct (interpretation)	Based on total scores		Based on error scores	
	F_1_ (executive function)	F_2_ (working memory)	ICA_1_ (focused attention)	ICA_2_ (short-term spatial memory)
Annual change in percentage points	− 0.92	− 0.75	− 0.36	− 0.05
(95% CI)	(− 0.98; − 0.87)	(− 0.81; − 0.69)	(− 0.41; − 0.31)	(− 0.09; − 0.01)
Regression coefficients for a standardized outcome				
Age	− 0.06 (0.00)***	− 0.05 (0.00)***	− 0.03 (0.00)***	− 0.01 (0.00)**
Female	− 0.07 (0.04)	− 0.02 (0.04)	− 0.05 (0.04)	0.11 (0.04)*
Higher education	0.40 (0.04)***	0.38 (0.04)***	0.48 (0.04)***	− 0.04 (0.05)
Living without partner (widowed/ separated/ divorced/ single)	0.04 (0.03)	0.02 (0.04)	0.07 (0.04)	− 0.03 (0.04)
Working (full-time or part-time)	0.15 (0.04)***	0.10 (0.04)*	0.06 (0.05)	0.04 (0.05)
Diabetes	0.06 (0.08)	0.04 (0.09)	− 0.01 (0.10)	− 0.07 (0.11)
High blood pressure	− 0.05 (0.04)	− 0.08 (0.04)	− 0.14 (0.04)**	− 0.13 (0.05)**
Heart disease	− 0.13 (0.07)	− 0.10 (0.07)	− 0.11 (0.08)	− 0.03 (0.08)
Stroke	− 0.34 (0.10)**	− 0.38 (0.11)***	− 0.47 (0.12)***	− 0.11 (0.13)
Ever experienced a head injury where you lost consciousness	0.08 (0.05)	0.02 (0.05)	0.10 (0.05)	0.07 (0.06)
Problems with hearing	− 0.02 (0.04)	− 0.03 (0.04)	0.04 (0.04)	− 0.06 (0.04)
Depression	− 0.07 (0.04)	− 0.09 (0.04)*	− 0.05 (0.05)	− 0.09 (0.05)
Isolation (no confiding relations since 16 years old)	− 0.20 (0.11)	− 0.13 (0.11)	0.11 (0.13)	− 0.00 (0.14)
Any physical activity lasting at least 20 min that has left you out of breath in the last month	0.02 (0.06)	0.06 (0.06)	0.04 (0.07)	0.07 (0.07)
Drinking alcohol more often than once a week	0.08 (0.03)*	0.10 (0.03)**	0.06 (0.04)	0.02 (0.04)
Exceeding two alcoholic drinks per occasion	− 0.10 (0.04)*	− 0.10 (0.04)*	− 0.10 (0.04)*	0.03 (0.05)
Current smoker	− 0.07 (0.07)	− 0.12 (0.07)	− 0.21 (0.09)*	− 0.05 (0.09)
Ever smoking	− 0.07 (0.03)*	− 0.08 (0.04)*	− 0.08 (0.04)*	0.01 (0.04)
BMI [ref Healthy (18.5–24.9)]				
Underweight (< 18.5)	0.02 (0.17)	− 0.10 (0.18)	0.04 (0.20)	0.11 (0.22)
Overweight (25.0–29.9)	− 0.03 (0.04)	− 0.03 (0.04)	− 0.12 (0.04)**	− 0.04 (0.04)
Obese (≥ 30)	0.05 (0.05)	0.06 (0.05)	− 0.07 (0.06)	− 0.07 (0.06)
Self-reported sleep dissatisfaction	0.01 (0.04)	0.01 (0.04)	0.02 (0.04)	− 0.15 (0.05)**

*p < 0.05; **p < 0.01; ***p < 0.00.

Age is a significant decrease predictor for all the latent constructs. Notably, the estimated effect of age is larger for outcomes based on total test scores than those based on error scores. This can be explained by the fact that total scores reflect the performance across multiple cognitive subsystems, all of which must remain unaffected for the total score to be preserved. In contrast, error scores capture specific aspects of cognitive functioning, which may not aggregate as rapidly with age.

Regression estimates demonstrate that female gender was positively associated with short-term spatial memory. Higher education was found to be advantageous across all cognition domains except for short-term spatial memory. Engaging in full-time or part-time employment had a slight positive effect on executive function and working memory. Among the health conditions considered, stroke was found to have a moderate adverse effect on three of the domains, and high blood pressure was detrimental to focused attention and short-term spatial memory. Surprisingly, consuming alcohol more frequently than once a week was associated with improved performance in both executive function and working memory. However, consuming more than two alcoholic drinks at a time was associated with decreased performance in executive function, working memory and focused attention. Furthermore, smoking and being overweight were found to negatively affect focused attention.

Table 3 indicates that predictors with the largest effect sizes for executive function, working memory and focused attention domains were higher education and stroke, whereas high blood pressure and sleep dissatisfaction were the most prominent for short-term memory. The effect sizes for all the latter predictors ranged from small to medium in magnitude.

Using a more conservative significance threshold of *p* < 0.01, the associations with age remained statistically significant for all the four domains. Likewise, higher education remained significantly associated with executive function, working memory, and focused attention, while stroke retained significant associations with executive function, working memory, and focused attention; stroke showed significant associations with executive function, working memory, and focused attention; high blood pressure and exceeding two alcoholic drinks per occasion remained significantly associated with focused attention; and ever smoking was significantly associated with executive function, working memory, and focused attention.

## Discussion

In this study, we conducted a comprehensive multivariate analysis of cognitive data collected at baseline from a digital cohort study. By leveraging the strengths of digital technology, we identified latent cognitive constructs, compared the effect of age across various cognitive domains, and evaluated the impact of dementia risk factors on cognitive function. The largest effect was observed in the executive function domain, with an estimated reduction of − 0.06 standard deviations (SD) per year of age. This was followed by working memory, which showed an effect of − 0.05 SD per year. Focused attention had an effect of − 0.03 SD annually, while short-term spatial memory showed the smallest effect of − 0.01 SD each year. Stroke was found to have a negative impact on executive function, working memory, and focused attention, while high blood pressure primarily affected focused attention and short-term spatial memory. Excessive alcohol consumption and smoking were harmful to working memory, executive function, and focused attention, and depression had an adverse effect on working memory. Previous studies on hypertension, stroke, and depression have reported associations with attentional and executive impairments^21–23^. However, the existing literature lacks sufficient consistency to identify clear links with specific cognitive tests. Sleep dissatisfaction was related to poorer short-term spatial memory. On the other hand, higher education was positively associated with performance in three of the four cognitive domains, and employment was beneficial for both working memory and executive function.

As the study is based on cross-sectional data, the reported effects of age reflect age-related differences between individuals rather than longitudinal within-person change.

Cognitive function naturally declines with age due to various physiological brain processes^24^. With advancing age, specific brain regions may experience diminished efficiency or functional decline. Age-related neuroanatomical brain changes were found to be unevenly distributed across different brain regions^25^. Specifically, the gray matter volume in regions such as the prefrontal cortex, medial temporal lobe, and hippocampus demonstrated the highest rate of reduction, whereas other regions, such as the cingulate gyrus and the occipital cortex were less affected^26^. Similarly, white matter integrity, which is linked to cognitive impairment^27^, declines unevenly across the brain^28^. Consequently, different aspects of cognition may deteriorate at varying rates with advancing age^29^.

Our findings are consistent with this assumption. The annual cognitive decline rates we observed align with those reported in other studies. In^30^, the age-related decrease in the memory domain was assessed at − 0.038 SD per year, while executive function showed a decrease of − 0.030 SD per year.

For comparison^31^, concluded that many fluid cognitive abilities, particularly psychomotor skills and processing speed, reach their peak in the third decade of life, followed by a decline at an estimated rate of − 0.02 SD per year. Similarly^32^, reported domain-specific rates of decline between ages 70 and 82, with processing speed showing the greatest decrease (− 0.088 SD per year), followed by visuospatial ability (− 0.054 SD per year), memory (− 0.028 SD per year), and verbal ability (− 0.003 SD per year). Our estimates of age effects in cognitive domains are consistent with those reported in other studies, although the effect on executive function appears to be larger than the decline reported in healthy aging populations. One possible explanation for this discrepancy is that our analysis utilized cross-sectional data, whereas the studies we referenced employed longitudinal data. Consequently, the observed age-related associations may be influenced by cohort effects, such as differences in familiarity with digital technologies, rather than reflecting solely biological aging. On the other hand, cross-sectional data are not prone to practice effects, where repeated testing can lead to artificially improved scores, masking true declines. Additionally, longitudinal studies often experience selective attrition, with individuals showing greater cognitive decline more likely to drop out over time, potentially leading to underestimation of the effect of age. Future research employing longitudinal designs to track individual cognitive trajectories is essential for developing more precise prognoses of cognitive decline and understanding how various dementia risk factors influence these trajectories.

Investigating cognitive abilities through test batteries is well established, with numerous studies using similar tests through clinical assessments^33]– [34^ or online platforms^16^. Our study is unique in that it incorporates intermediate outcomes such as error counts as primary data, which have not been commonly used in previous analyses. Our findings suggest that incorporating such data enables a more detailed analysis. Relying solely on total scores may not provide sufficient insight, particularly for individuals transitioning through different age stages. The incorporation of measures such as reaction time, as used in the FLAME battery^15^, could better address these considerations and increase the accuracy of cognitive evaluations.

We applied the blind signal separation method Independent Components Analysis (ICA) to the error data, isolating two statistically independent signals. On the basis of their loading patterns, we interpreted these signals as representing focused attention and short-term spatial memory. While these domains are governed by distinct brain regions^35^, they are likely to interact to some extent. Nonetheless, we assumed that the connection between them is not strong enough to clearly manifest in error-based measures.

In this study, we identified several well-established dementia risk factors as statistically significant and quantified their effects on different domains. These findings provide additional support for the use of these cognitive tests to validate age-related changes and risk factors, confirming the patterns observed in prior research and enhancing their concurrent validation as useful tools for broader applications. These domain-specific patterns may provide a useful starting point for developing targeted interventions aimed at supporting cognitive health in aging populations.

Some of our findings are controversial. For example, frequent alcohol consumption was positively associated with cognitive outcomes, which persisted even after adjusting for health conditions (data not shown). One possible explanation is a potential confounding effect that we could not fully control for with the available data. Another possible explanation is selection bias, as the study population consists of self-enrolled online volunteers who are likely more health-conscious, cognitively intact, and socioeconomically advantaged than the general population. As a result, individuals reporting frequent alcohol use in this sample may differ systematically from similar individuals in the broader population—for example, reflecting moderate social drinking within healthier lifestyles rather than problematic use. The association between alcohol use and cognitive outcomes should therefore be verified in more representative cohorts. This is important, as other studies have reported similar patterns^16^. Conversely, consuming larger amounts of alcohol (more than two drinks at a time) had a pronounced negative impact on multiple domains.

A more thorough analysis of confounders is necessary for a more precise evaluation of the impact of risk factors. Nevertheless, for the purposes of this study, we deliberately chose to focus on demographic confounders without delving into which specific variables to adjust for each risk factor. This decision was made to maintain a streamlined process, allowing for a broader yet efficient, overview of potential associations before committing to a more granular and in-depth analysis.

This study highlights the value of incorporating detailed and advanced assessment methods to better understand cognitive function and its decrease with age. The ability to confirm these patterns against established findings demonstrates the promise of digital cognitive assessments as reliable tools. The scalability of these methods for use in large populations offers a unique opportunity to expand cognitive testing across diverse groups. Future research should continue to refine these approaches to provide a clearer picture of cognitive aging and its associated risk factors. The potential usefulness of these findings is to help inform targeted interventions to delay or prevent cognitive decline in the elderly population.

The primary limitation of this study is the nonrandomized nature of the cohort, which introduces a risk of selection bias. Specifically, there was a disproportionate representation of younger women and too few individuals with high levels of alcohol consumption and those who engaged in minimal physical activity.

Data collected online may lack the precision of traditional clinical assessments because of differences in testing conditions. In-clinic assessments occur in controlled settings with professional supervision, minimizing distractions. In contrast, online assessments are completed at home, where environmental variability, device differences, and lack of supervision can introduce measurement noise. These factors may affect the precision of the data.

Participation in this study required individuals to self-register and complete tasks online, which may limit generalizability to those more comfortable with digital technology. This requirement could lead to selection bias, particularly among older adults. However, Norway ranks among the highest in Europe for digital access and competence. In 2023, 93% of households had access to a personal computer, and 81.1% of individuals aged 16–74 reported at least basic digital skills^36–38^. Furthermore, the PROTECT platform includes robust quality control measures, such as data screening for technical issues and participant support, which help enhance data reliability. Nonetheless, the study sample may still represent a more digitally engaged subgroup, a factor that should be considered when interpreting the results. Broader generational trends, such as the Flynn effect^39,40^, may also influence the age-related patterns observed. The Flynn effect refers to population-wide increases in cognitive test scores over time. These changes are potentially driven by factors like improvements in education, health, or technology exposure. These trends can affect normative baselines and complicate comparisons of cognitive scores across different cohorts.

The relatively small number of participants reporting certain conditions may affect the generalizability of some findings. This is partly due to participants having the option to skip questions about medical diagnoses and partly because not all questionnaires were completed. Formal correction for multiple testing across all predictor–outcome models was not applied. Instead, to highlight robust associations, we considered a more stringent significance threshold of *P*-value < 0.01, which approximates a partial adjustment across the four outcome domains but does not account for the number of predictors. Accordingly, the results should be interpreted with appropriate caution. While our study provides preliminary evidence, further longitudinal research is necessary to validate these findings and better understand their implications.

### Strengths and limitations

Strengths: Our study offers valuable insights into cognitive aging by leveraging a digital cohort, providing a unique perspective on cognitive function in a broad, preclinical context.

Limitations: Limitations of the study include its cross-sectional design, potential selection bias due to nonrandomized sampling, and lack of correction for multiple comparisons.

## Conclusion

Our study underscores the utility of online cognitive assessments, particularly in their ability to identify cognitive domains, assess the effects of age, and evaluate the impact of dementia risk factors. Online assessment of aging participants before noticeable cognitive decline allows for the detection of subtle signals and new insights from the rich data collected, providing a valuable tool for both research and clinical practice. This study demonstrates that such digital assessments offer a scalable, cost-efficient solution for broader participation in cognitive screening. By enhancing accessibility and engagement, online tools provide a promising framework for early intervention and prevention in aging populations, advancing our understanding of cognitive decline and dementia.

## Supplementary Information

Below is the link to the electronic supplementary material.

Supplementary Material 1

## Data Availability

The raw data supporting the conclusions of this article will be made available from the corresponding author on reasonable request, without undue reservation.
