# Supplementary material for: Discerning cognitive domains through online assessment in aging participants in Norwegian Cohort 50+
Source: Sci Rep. 2025 Nov 27;15:42357. doi: 10.1038/s41598-025-26437-8 (PMC12660784; doi:10.1038/s41598-025-26437-8)
Supplement: Supplementary file 1 — Supplementary Material 1 [file 41598_2025_26437_MOESM1_ESM.docx]

Supplementary Table S1. Overview of Cognitive Tests Included in the Study's Cognitive Test Battery.

| **Test** | **Measuring what** | **Duration** | **Short description** | **Error score details** |
| --- | --- | --- | --- | --- |
| *Digit Span* | aspects of attention | Max 3 min | Participants are presented with a sequence of digits, which they must recall in the same order or in reverse order. Each successful trial is followed by a sequence that is one digit longer, and each unsuccessful trial is followed by a sequence that is one digit shorter. | For each sequence length, up to three trials are allowed, and the sequence length increases if the recall is correct. |
| *Paired Associates Learning* | Long term spatial memory | Max 3 min | A series of objects appear in different cells on the screen. The participant must remember the location of each object and, when prompted, click the correct cell. Each level allows three attempts. | Next level if no more than three errors were made. |
| *Self‐Ordered‐Search* | Short term spatial memory | Max 5 min | A series of boxes appear on the screen, with one containing a diamond. The participant clicks boxes to find the diamond, which then moves to a new box. They must avoid selecting a box where the diamond was previously found. Efficiently locating the diamond results in higher scores. | This task has a ratchet-style approach in which each successful trial is followed by a new sequence that is longer. Runs until failure, with three wrong attempts in same stage. |
| *Grammatical Reasoning task* | verbal/grammatical reasoning | 3 min | A sentence appears below a circle and square on the screen. The participant must decide whether the sentence correctly describes the positions of the shapes. | Error scores are defined as the total number of incorrect responses |
| *Switching Stroop* | Executive function and impulsivity | Max 3 min | Participants were presented with a colored block and two colored words in separate boxes, along with cue text in each corner. Based on the cue:   - If it said "color," participants selected the word matching the block's hue. - If it said "word," they chose the word that described the block's color.   This task required participants to alternate between these rules, effectively testing their cognitive flexibility and attention. | Error scores are defined as the total number of incorrect responses |
| *Trail Making A and B* | Visual attention and cognitive flexibility | Max 3 min | Participants are instructed to connect a series of 25 dots in the correct order as quickly as possible.  Trail Making Test A involves only numbers (1 to 25), while Trail Making Test B alternates between numbers and letters (1 – A – 2 – B – 3 – C – etc.).  In the PROTECT study, the Trail-Making Test A and B were combined into one task where participants connected dots in the correct order as quickly as possible. The task had four stages of increasing difficulty:  Trial 1 (C1): Connect numbers from 1 to 8 in ascending order.  Trial 2 (C2): Connect numbers from 1 to 25 in ascending order.  Trial 3 (C3): Alternate between numbers and letters in ascending order, starting from 1-A-2-B to 4-D.  Trial 4 (C4): Similar to Trial 3 but with a larger range, going from 1-A-2-B to 13-L.  The time taken to complete Trials A and B is recorded.  A time-out occurs if a trial is not completed within 30 seconds. | Error scores are defined as the total number of incorrect responses |
